# Functional connectivity during a social emotion task in adolescents and in adults

**DOI:** 10.1111/j.1460-9568.2009.06674.x

**Published:** 2009-03

**Authors:** Stephanie Burnett, Sarah-Jayne Blakemore

**Affiliations:** Institute of Cognitive Neuroscience, University College London17 Queen Square, London WC1N 3AR, UK

**Keywords:** functional integration, medial prefrontal cortex, mentalising network, PPIs, social brain, social cognition, theory of mind

## Abstract

In this fMRI study we investigated functional connectivity between components of the mentalising system during a social emotion task, using psychophysiological interaction (PPI) analysis. Ten adults (22–32 years) and 18 adolescents (11–18 years) were scanned while thinking about scenarios in which a social or a basic emotion would be experienced. Unlike basic emotions (such as disgust and fear), social emotions (such as embarrassment and guilt) require the representation of another’s mental states. In both adults and adolescents, an anterior rostral region of medial prefrontal cortex (arMPFC) involved in mentalising showed greater connectivity with the posterior superior temporal sulcus (pSTS) bordering on the temporo-parietal junction (TPJ) and with anterior temporal cortex (ATC) during social than during basic emotion. This result provides novel evidence that components of the mentalising system interact functionally during a social emotion task. Furthermore, functional connectivity differed between adolescence and adulthood. The adolescent group showed stronger connectivity between arMPFC and pSTS/TPJ during social relative to basic emotion than did the adult group, suggestive of developmental changes in functional integration within the mentalising system.

## Introduction

The mentalising system, comprising anterior rostral medial prefrontal cortex (arMPFC), posterior superior temporal sulcus (pSTS) bordering on the temporo-parietal junction (TPJ) and the anterior temporal cortex (ATC), is activated when participants reflect on mental states such as intentions, beliefs and desires. Although the mentalising system is referred to as a network (Frith & Frith, 2003), and its constituent regions are connected anatomically in macaques (Bachevalier *et al.*, 1997; Barbas *et al.*, 1999), it is unknown whether these brain regions show functional connectivity during mentalising tasks. The first aim of the current study was to investigate functional connectivity within the mentalising system during a social emotion task.

Social emotions such as embarrassment and guilt require the representation of another person’s mental states, whereas this is not the case for basic emotions such as fear and disgust. In our social emotion task, participants silently read a series of scenarios designed to evoke social emotions (embarrassment or guilt) or basic emotions (disgust or fear). Using conventional analysis, we found that the mentalising system showed greater mean activity during social than during basic emotions (Burnett *et al.*, 2008). Our first aim here was to extend this finding by testing the hypothesis that functional connectivity within the mentalising system would be higher during social than basic emotions. Specifically, using psychophysiological interaction (PPI) analysis, we tested the prediction that activity within arMPFC would more strongly predict activity in pSTS/TPJ and ATC in social than in basic emotions.

The second aim was to investigate how functional connectivity within the mentalising system changes with age. Recent developmental functional magnetic resonance imaging (fMRI) studies of social cognition using conventional analyses (including our previous study; Burnett *et al.*, 2008) have demonstrated a shift in activity within the mentalising system between late childhood and adulthood. Activity in arMPFC tends to decrease with age, while activity within temporal regions of the mentalising system shows the opposite developmental pattern (see Blakemore, 2008; for a meta-analysis). In the current study, we hypothesised that there would also be a developmental shift in functional connectivity within the mentalising system. This hypothesis was based on two findings. First, the number of synapses in prefrontal cortex (PFC) changes during adolescence (Huttenlocher, 1979,Huttenlocher *et al*., 1982), as does myelination of axons in this region (Yakovlev & Lecours, 1967). Second, fMRI studies using non-social tasks have shown evidence for developmental changes in functional or resting-state connectivity, for example during the go/no-go task (Stevens *et al.*, 2007, 2009) and baseline (Fair *et al.*, 2007, 2008). These studies show that correlated activity within brain networks increases with age. However, as no previous study has investigated development of functional connectivity in social cognition tasks, it is unknown whether this would show a similar developmental trajectory.

PPI analysis is a statistical technique based on linear regression and provides insights that are independent and fundamentally different from those gained by conventional analysis. PPI analysis is based on the principle that if activity in one region (area A) predicts activity in another region (area B), then the strength of the prediction reflects the influence area A could be exerting on area B. If the strength of the prediction varies with the psychological context in which the physiological activity is measured (i.e. experimental condition) then this is evidence for a psychophysiological interaction (Friston *et al.*, 1997). In PPI analysis, a brain region of interest is defined as the physiological source. We defined our source region as arMPFC because of its involvement in mentalising (Amodio & Frith, 2006), and tested for task-dependent correlations between activity in arMPFC and activity elsewhere within the mentalising system.

## Materials and methods

### Participants

The participants were 18 adolescents (11.40–18.17 years; mean, 15.03 years), and 10 adults (22.92–31.83 years; mean, 26.41 years), with no history of psychiatric or neurological disorder. Mean full-scale IQ (FSIQ), as measured by the Wechsler Abbreviated Scale of Intelligence (WASI; Harcourt Assessment, Inc., 1999) did not differ between adult (mean ± SD, 111.14 ± 14.10) and adolescent (115.52 ± 6.63) groups (independent samples *t*-test, *t*_22_ = −1.052, *P*>0.3). Three adult participants did not complete the WASI but as they had completed university-level education their IQ level was judged to be similar to that of the other participants. Written informed consent was obtained prior to the study from all adult participants, and from a parent or guardian of participants younger than 18. The study was approved by the UCL National Hospital for Neurology and Neurosurgery Ethics Committee and carried out according to the Declaration of Helsinki.

In this study participants were all female, in consideration of the significant sex differences which have been reported in various measures of brain anatomy, including within brain regions involved in emotion and social cognition (Lenroot *et al.*, 2007; Schmithorst *et al.*, 2008), the sex differences reported in fMRI studies of emotion processing in adolescents and in adults (Yurgelun-Todd *et al.*, 2002; Hall *et al.*, 2004; Killgore & Yurgelun-Todd, 2004; Moll *et al.*, 2005), and finally the documented sex differences in the unfolding time-course of adolescent neuroanatomical development (Giedd *et al.*, 1999).

### Experimental design

Participants underwent fMRI scanning as they read sentences describing emotional scenarios. A total of 554 functional volumes were acquired over two consecutive scanning sessions lasting 12 min each. In this PPI analysis, the experiment had a 2 × 2 mixed factorial design, comprising between-subjects factor group (adolescent vs. adult) and within-subjects factor emotion (social vs. basic). Note that in our previous study we included a second within-subjects factor (self vs. other): scenarios featured either the participant or another person (the participant’s mother). However, as there were no differences in activity within the mentalising system for the main effect of self vs. other, and in order to increase power, we collapsed across self and other scenarios in the PPI analysis.

The social emotion scenarios featured either embarrassment or guilt, and the basic emotion scenarios featured disgust or fear. Examples of social emotion sentences are ‘You were at the cinema with your friend and you got loud hiccups’ (embarrassment) and ‘You laughed when your friend told you she was feeling upset’ (guilt). Examples of basic emotion sentences are ‘You saw a big hairy fly laying eggs in your friend’s lunch’ (disgust) and ‘A dog was growling and trying to bite you and your friend’ (fear). The emotion sentences were designed to maximize the difference between social and basic emotion conditions in terms of the requirement for mentalising. Therefore, basic emotion sentences featured immediate, visceral disgust- or fear-evoking situations. Both social and basic scenarios featured the protagonist plus one other person. This ensured that the difference between the social and basic emotion conditions was the need to take into account another person’s mental state, not the mere presence of another person in the scenario. Half of the emotion sentences were in the first-person perspective (the protagonist was ‘you’), and half were in the third-person perspective (the protagonist was ‘your Mum’). We equated the mean (plus the range) word length and the number of clauses between conditions.

Sentences were presented in blocks, with three sentences per block. Participants had 9 s to silently read each sentence, imagine the scenario and rate their imagined emotional response on a discrete rating scale from 1 (I would not feel the emotion at all) to 4 (I would feel the emotion very much). The experiment was blocked by emotion such that, within a block, all three scenarios featured the same emotion (disgust, embarrassment, fear or guilt). At the start of each block, a 1-s cue screen informed participants which emotion the proceeding three sentences would feature.

Each 12-min session of the fMRI experiment contained 24 emotion blocks, lasting 28 s each. Condition order was fully randomised. In addition there were two 28-s visual fixation blocks per session, occurring one-third and two-thirds of the way through each of the two sessions. Stimulus presentation was programmed in Cogent (http://www.vislab.ucl.ac.uk/Cogent/index.html) running in Matlab 6.5, which recorded participant responses.

### Imaging data acquisition

A 1.5 T Siemens Sonata head MRI scanner was used to acquire both 3-D T_1_-weighted fast-field echo structural images and multi-slice T_2_*-weighted echo-planar volumes with blood oxygenation level-dependent (BOLD) contrast. Each functional brain volume was composed of 33 3-mm axial slices with a 1.5-mm gap and in-plane resolution of 3 × 3 mm, angled at 30 ° to cover the whole brain and minimize signal dropout from the facial sinuses. Repetition time was 3 s. Functional data were acquired in two scanning sessions of ∼ 12 min each, in which a total of 554 volumes were acquired, or 277 scans per session. The acquisition of a T_1_-weighted anatomical image occurred after the two functional scanning sessions for each participant. The total duration of scanning was ∼ 35 min per participant.

### Conventional imaging data analysis

As reported in Burnett *et al.* (2008), fMRI data were analysed by collapsing the four emotions disgust, embarrassment, fear and guilt into two emotion conditions, social and basic. This was because our hypothesis related to differential neural effects of social vs. basic emotion, not to the neural effects of specific emotions.

Analysis was conducted using SPM2 (http://www.fil.ion.ucl.ac.uk/spm). The first six functional image volumes from each run were discarded to allow for T_1_ equilibrium effects, leaving 542 image volumes per participant. Pre-processing included rigid-body transformation (realignment) and slice timing to correct for head movement and slice acquisition delays. The images were stereotactically normalised into the standard space defined by the Montreal Neurological Institute (MNI) template using the mean of the functional volumes, and smoothed with a Gaussian filter of 6 mm full-width at half-maximum. The time series for each participant were high-pass-filtered at 128 s to remove low-frequency drifts.

The analysis of the functional imaging data entailed the creation of statistical parametric maps representing a statistical assessment of hypothesised condition-specific effects (Friston *et al.*, 1994), which were estimated with the General Linear Model. The effects of interest were the two scenario block types: social emotion and basic emotion (results from the self vs. other factor are reported in detail in Burnett *et al.*, 2008 and will not be mentioned further here). We modelled the six realignment parameters as effects of no interest, in order to account for possible group differences in head movement. Each component of the model then served as a regressor in a multiple regression analysis for each participant. The resulting parameter estimates for each regressor at each voxel were then entered into a second level analysis where ‘participant’ served as a random effect in a within-subjects anova.

Main effects and interactions between conditions were specified by appropriately weighted linear contrasts, and determined using the *t*-statistic on a voxel-by-voxel basis. Statistical analysis at the second level was performed for each group separately to examine the main effects of emotion (social > basic). To compare directly group differences in activation to emotion we looked at two-way interaction between group and emotion using the appropriate contrasts (for further details, see Burnett *et al.*, 2008).

### PPI analysis

PPI analysis assesses the hypothesis that activity in one brain region can be explained by an interaction between the presence of a cognitive process and activity in another part of the brain. We used PPI analysis to estimate functional connectivity between a source (arMPFC) and target regions of interest (pSTS/TPJ, ATC), during social vs. basic emotion. Our previous conventional fMRI analysis (Burnett *et al.*, 2008) revealed activity to social vs. basic emotion within arMPFC in the adult and adolescent groups. We also observed activity in parts of pSTS/TPJ in both groups, and in left ATC in the adult group only. The selection of arMPFC as the PPI source region denoted activity within arMPFC as the physiological regressor in the PPI analysis. Emotion condition (social vs. basic) was the psychological regressor. A third regressor in the analysis represented the interaction between the first and second regressors.

The precise region of arMPFC from which the physiological regressor (BOLD signal change) was extracted was defined in the following manner. First, taking as our reference the mentalising region of arMPFC (BA 10) in Gilbert *et al.* (2007), we defined arMPFC as the volume from −8 to +8 on the *x*-axis, from +40 to +56 on the *y*-axis and from −12 to +30 on the *z*-axis. Then, in each single-subject *t*-contrast map for the emotion contrast (social > basic), thresholded at *P*<0.005 uncorrected (minimum voxel extent 4), we used SPM2 to locate the nearest local maximum to the centre of this volume (i.e. the nearest single-subject peak to the co-ordinate [0 48 9]). We then created a volume of interest in the form of a sphere of radius 8 mm centred on the single-subject peak. If there was no significantly active cluster within arMPFC at this threshold (*n =*8 datasets), we lowered the threshold to *P*<0.05 uncorrected (minimum voxel extent 4). Two datasets that did not contain a peak within our defined arMPFC volume at this significance level were excluded (one adolescent, one adult), leaving 17 adolescent and nine adult datasets in the subsequent PPI analysis. Finally, we smoothed the single-subject social vs. basic *t*-contrast maps to facilitate group level analysis, and extracted the BOLD signal time series from each subject’s volume of interest in arMPFC.

Voxel-wise PPI analysis was conducted at the combined group level (*n*=26), in order to identify target brain regions that showed a significant increase in functional coupling with arMPFC during social relative to basic emotion, at a threshold of *P*<0.05 (minimum voxel extent 4) with family-wise error correction, except within *a priori* target regions of interest where an uncorrected threshold of *P*<0.01 (minimum voxel extent 4) was applied (Penny *et al.*, 2003). *A priori* target regions were defined as components of the mentalising system, that is, pSTS/TPJ (co-ordinates as in Aichhorn *et al.*, 2006) plus the caudal portion of STS that extends into TPJ (Frith & Frith, 2003; Hein & Knight, 2008) and the ATC (Frith & Frith, 2003; Burnett *et al.*, 2008; Hein & Knight, 2008). Voxel-wise PPI analysis was conducted within each age group separately, and to directly compare group differences in functional connectivity we looked at the interaction between the PPI (interaction between arMPFC activity and emotion) and group (adolescent vs. adult). Using planned *t*-contrasts we then investigated, within each group separately, functional connectivity during social vs. basic emotion within brain regions that showed this group-by-PPI interaction. Finally, we repeated the group-by-PPI analysis with FSIQ as a covariate of no interest (FSIQ scores were not available for all participants, resulting in *n*=6 adults and *n*=16 adolescents), to ascertain whether developmental differences in functional connectivity in the mentalising system could be explained by IQ differences.

## Results

### Behavioural data

Participants rated to what extent the protagonist of each scenario would feel a given emotion, on a discrete rating scale from 1 (not at all) to 4 (very much). Mixed-design repeated-measures 2 × 2 anova [between-groups factor: group (adolescent, adult); within-group factor: emotion (social, basic); after exclusion of two datasets as outlined in Methods] on single subject mean social and basic emotion ratings across the whole experiment showed a significant main effect of emotion (*F*_1,24_ = 6.73, *P*=0.02, partial *η*^2^ = 0.22). Specifically, basic emotion scenarios were given higher ratings than social emotion scenarios (*t*-test: *t*_25_ = −2.46, *P*=0.02). There was no main effect of group (*F*_1,24_ = 0.35, *P*=0.56), nor was there a significant interaction between age group and emotion (*F*_1,24_ = 0.75, *P*=0.39; see Table 1).

**Table 1:** Emotion ratings by group and emotion condition (*n*= 26)

Group	Rating	*n*
Social		
Adult	3.069 ± 0.301	9
Adolescent	3.041 ± 0.364	17
Basic		
Adult	3.291 ± 0.364	9
Adolescent	3.152 ± 0.407	17

Data are presented as means ± SD.

### Psychophysiological interaction data

#### Psychophysiological interaction across subjects

**Fig. 1:**
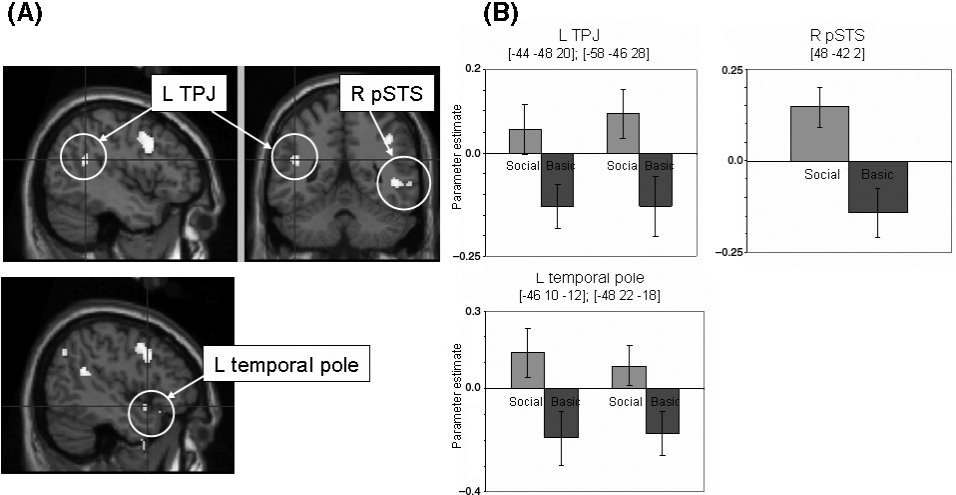
PPI results for all subjects: (A) regions of significant interaction between emotion (social vs. basic) and activity in arMPFC, shown at *P*< 0.01 projected onto transverse and sagittal T1 images; (B) graphs showing parameter estimates for regions of significant interaction between emotion (social vs. basic) and activity in arMPFC.

Across subjects (*n*=26, aged 11 to 32), PPI analysis revealed a significant interaction between emotion (social > basic) and arMPFC activation, expressed in regions of the mentalising system. Specifically, the left TPJ, right pSTS and left ATC showed greater functional connectivity with arMPFC during social than during basic emotion scenarios (Table 2; Fig. 1). No other regions showed significant interaction with arMPFC during social vs. basic emotions.

**Table 2:** PPI results: brain regions expressing an interaction between activity within arMPFC and emotion condition (social vs. basic) across subjects (*n*=26)

Region and MNI co-ordinates	*P*-value	*T*-value	*Z*-value	Size in voxels at *P*<0.01
R pSTS				
[48 −42 2]	< 0.001	4.21	3.63	343
[60 −42 2]	0.001	3.62	3.21	(part of above)
[62 −32 −4]	0.002	3.21	2.91	(part of above)
L TPJ				
[−44 −48 20]	0.002	3.25	2.94	51
[−42 −46 28]	0.007	2.66	2.48	(part of above)
[−58 −46 28]	0.003	2.96	2.71	10
[−46 −66 38]	0.005	2.76	2.56	8
L ATC				
[−46 10 −12]	0.005	2.75	2.55	14
[−46 8 −12]	0.006	2.7	2.51	7
[−66 −4 −22]	0.003	3.08	2.81	50
[−48 22 −18]	0.005	2.77	2.56	6

R, right; L, left.

#### Psychophysiological interaction within each group separately

Within the adult group (*n*=9, aged 22 to 32), PPI analysis revealed a significant interaction between emotion (social > basic) and arMPFC activation, expressed in the ATC bilaterally and in right pSTS/TPJ (Table 3). That is, in the adult group these regions showed greater functional connectivity with arMPFC during social than during basic emotions.

**Table 3:** PPI results: brain regions expressing an interaction between arMPFC activity and emotion condition (social vs. basic), within each group separately

Region and MNI co-ordinates	*P*-value	*T*-value	*Z*-value	Size in voxels at *P*<0.01
Adult (*n*= 9)				
R pSTS/TPJ				
[62 −44 4]	0.002	4.12	2.93	71
[68 −44 4]	0.002	3.97	2.87	(part of above)
L ATC				
[−48 16 −20]	0.002	3.99	2.88	31
R ATC				
[58 6 −32]	0.003	3.83	2.81	8
Adolescent (*n*= 17)				
L pSTS extending into L TPJ				
[−36 −36 8]	0.001	3.93	3.24	402
[−48 −38 8]	0.001	3.69	3.09	(part of above)
[−44 −44 0]	0.002	3.47	2.95	(part of above)
L TPJ				
[−58 −48 26]	0.006	2.86	2.53	8
R pSTS/TPJ				
[60 −30 −4]	0.001	3.64	3.06	249
[36 −28 18]	0.001	3.62	3.05	(part of above)
[60 −38 0]	0.003	3.15	2.74	(part of above)
L ATC				
[−44 −4 −24]	0.003	3.1	2.7	11

R, right; L, left.

Within the adolescent group (*n*= 17, aged 11 to 18), PPI analysis revealed a significant interaction between emotion (social > basic) and arMPFC activation, expressed in the left pSTS extending into left TPJ, in left TPJ proper, in right pSTS and in the left ATC (Table 3). That is, in the adolescent group these regions showed greater functional connectivity with arMPFC during social than during basic emotions.

#### Interaction between emotion and group

In our conventional analysis of the fMRI data (Burnett *et al.*, 2008), we found that mean activity within certain regions of the mentalising system differed as a function of age group (adolescent vs. adult). Specifically, adolescents showed stronger mean activity within arMPFC to social vs. basic emotion than did adults, whereas adults showed stronger mean activity within left ATC for this contrast compared to adolescents. In the current PPI analysis, we sought to determine whether task-dependent functional connectivity between arMPFC and other regions of the mentalising system also varied as a function of age group.

**Fig. 2:**
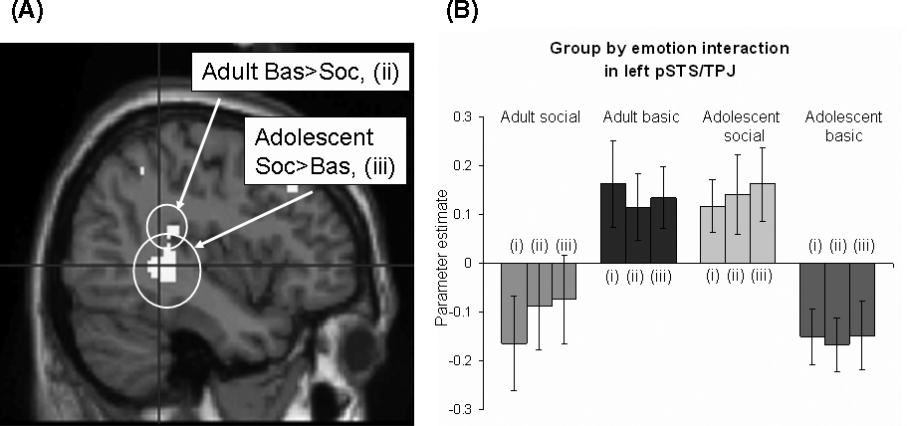
Interaction between group, emotion and arMPFC activation in left pSTS/TPJ. (A) Region showing significant interaction between group (adolescent vs. adult), emotion (social vs. basic) and activity in anterior rostral MPFC, shown at *P*< 0.01 projected onto a sagittal T1 image. Crosshair is at peak voxel [−40 −42 2]. Regions in which there was also a main effect of social vs. basic emotion within each group separately are circled. (B) Graph showing parameter estimates for this interaction. Key: (i) peak voxel [−40 −42 2]; (ii) secondary peak voxel [−44 −34 10]; (iii) secondary peak voxel [−38 −34 20].

Our analysis revealed a significant interaction between age group and the PPI between emotion condition and arMPFC activity, expressed within a region of left pSTS extending into TPJ (Table 4; Fig. 2A). In other words, functional connectivity between left pSTS/TPJ and arMPFC during social vs. basic emotion differed between adolescent and adult groups (Fig. 2B). *Post hoc* tests revealed greater functional connectivity in adolescent social relative to basic emotion between arMPFC and the central portion of left pSTS/TPJ {region (iii) in Fig. 2 [−44 −34 10]; adolescents: paired *t*-test, *t*_16_ = −1.83, *P*=0.04; adults: paired *t*-test, *t*_8_ = −0.76, *P*=0.24}. In a more peripheral portion of the left pSTS/TPJ region, adults showed the opposite pattern (region ii in Fig. 2 [−38 −34 20]; adults: paired *t*-test, *t*_8_ = −2.31, *P*=0.02; adolescents: paired *t*-test, *t*_16_ = −1.15, *P*=0.13). No other regions showed a significant interaction between group and condition with arMPFC. These results remained the same when FSIQ was entered as a covariate of no interest (left pSTS/TPJ peak: [−42 −44 4], *Z*=3.48, *P*<0.001, size in voxels at *P*<0.01 = 250; secondary peak: [−46 −38 12], *Z*=3.33, *P*<0.001; in addition, the primary and secondary peaks from the non-FSIQ covariate analysis were active above threshold), suggesting that this developmental difference in functional connectivity in the mentalising system is not explained by IQ differences between participants.

**Table 4:** PPI results: brain regions expressing an interaction between arMPFC activity, emotion condition (social vs. basic) and group (adolescent vs. adult)

Region and MNI co-ordinates	*P*-value	*T*-value	*Z*-value	Size in voxels at *P*<0.01
L pSTS extending into L TPJ				
[−40 −42 2]	< 0.001	3.75	3.29	247
[−44 −34 10]	0.001	3.45	3.08	(part of above)
[−38 −34 20]	0.003	2.97	2.72	(part of above)

**Fig. 3:**
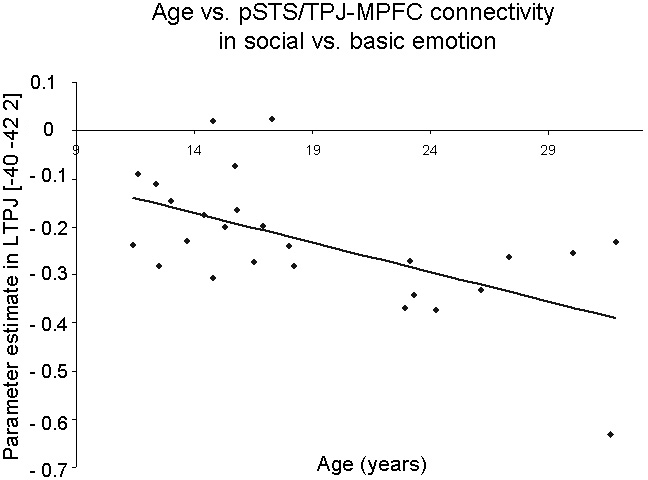
Negative correlation between age and left pSTS/TPJ–arMPFC connectivity during social relative to basic emotions for illustrative purposes.

Linear regression between participant age and connectivity with arMPFC during social vs. basic emotion revealed significant activity within a region of left pSTS/TPJ (peak: [−42 −42 0], *T*=3.55, *Z*=3.15, *P*=0.001, size in voxels at *P*<0.01 = 80), which overlapped with the region found to be active in the group × condition interaction. Fig. 3 shows single-subject PPI parameters representing functional connectivity between the arMPFC source region and activity in the peak voxel within left pSTS/TPJ for which there was a significant group (adolescent vs. adult) × emotion (social vs. basic) interaction, plotted against age in years.

## Discussion

The current study had two aims. The first aim was to investigate functional connectivity within the mentalising system during a social emotion task, using PPI analysis. Our previous conventional analysis showed that components of the mentalising system, namely arMPFC, pSTS/TPJ and ATC, were more active during social than during basic emotion processing (Burnett *et al.*, 2008). In the current study, we additionally found that these regions showed greater functional connectivity during social than during basic emotions. To our knowledge, this is the first study to demonstrate functional connectivity within the mentalising system.

The second aim of our study was to investigate how functional connectivity within the mentalising system changes with age. In our previous study, we found that activity in arMPFC during social relative to basic emotions was greater in adolescents than in adults (Burnett *et al.*, 2008). Here, we add to this developmental picture by showing that functional connectivity between arMPFC and left pSTS/TPJ is stronger during social relative to basic emotion processing in adolescents than it is in adults.

### Functional connectivity within the mentalising system

Conventional neuroimaging analysis provides information about how mean activity within a set of brain regions differs between conditions and between groups. PPI analysis differs in that it is used to evaluate the correlation of (non-averaged) activity between brain regions, and how this correlation differs as a function of the psychological context (experimental condition; Friston *et al.*, 1997). This correlation is taken to imply that the brain regions interact more during one experimental condition than during another.

Our previous conventional analysis showed that components of the mentalising network were active during social relative to basic emotions (Burnett *et al.*, 2008). The current PPI analysis revealed that components of the mentalising network also show functional connectivity during social relative to basic emotions. Specifically, bilateral pSTS/TPJ and left ATC show greater functional connectivity with arMPFC during social emotions than during basic emotions. This adds weight to the notion that these brain regions function as a network during mentalising tasks. It is possible that, at a neurophysiological level, the functional connectivity is mediated by direct anatomical connections between arMPFC and both pSTS/TPJ and ATC, as have been identified in rhesus macaques (Bachevalier *et al.*, 1997; Barbas *et al.*, 1999).

PPI analysis revealed functional connectivity between arMPFC and parts of the left ATC in both adult and adolescent groups. Our previous conventional analysis indicated that adults activated left ATC for social relative to basic emotion, whereas adolescents showed no such significant activation in this region. As conventional and PPI analyses test fundamentally different processes, in this case the two sets of results provide complementary information. The finding that, in the adolescent group, ATC showed functional connectivity with arMPFC during social relative to basic emotions, but was not found to be active in this contrast in the conventional analysis (i.e. no difference in mean activity during social vs. basic emotions), suggests a modulatory relationship between ATC and the arMPFC that is expressed in a task-dependent manner. This finding mirrors previous PPI studies, in which functional connectivity between brain regions is demonstrated in situations in which the same regions are not active in a particular contrast under conventional analysis. For example, Rao *et al.* (2008) showed that, whereas PFC was not active during an executive sensorimotor task, there was nevertheless evidence for functional connectivity between PFC and relevant sensorimotor regions during task performance; this implies that PFC plays a regulatory rather than a direct role in the task. Thus, one possibility in the current study is that, during social relative to basic emotions, arMPFC and ATC are engaged in a regulatory relationship. Further studies are needed to investigate the directionality of this relationship and explore in more detail its functional role.

### Developmental differences in functional connectivity

Our second aim in this study was to investigate whether functional connectivity within the mentalising system differed between the adolescents and adults. To our knowledge, no previous fMRI study has examined age differences in functional connectivity during a mentalising task. In the current study, we found evidence for an age-related decrease in functional connectivity between arMPFC and left pSTS/TPJ during social relative to basic emotions. This finding is at odds with the small number of developmental studies of functional connectivity in the literature, which report age-related increases in correlated activity within neural networks. However, all previous studies have been restricted to non-social domains. For example, functional connectivity has been investigated in adolescents vs. adults during go/no-go tasks (Stevens *et al.*, 2007, 2009), and static (rather than functional) connectivity has been investigated in adolescents vs. adults during resting baseline (Fair *et al.*, 2007, 2008; Kelly *et al.*, 2009). No previous study has investigated the development of functional connectivity during a social cognition or mentalising task.

### Laterality in pSTS/TPJ

In the current study, we report a group × condition interaction in functional connectivity within left pSTS/TPJ. While both age groups showed functional connectivity between arMPFC and right pSTS/TPJ during social relative to basic emotions, only the adolescent group showed functional connectivity with left pSTS/TPJ. The reason for this laterality effect is unclear. As discussed in detail in Aichhorn *et al.* (2006), functional imaging studies of mentalising have reported uniquely left-lateralised pSTS/TPJ activity (Goel *et al.*, 1995), more heavily left- (Ruby & Decety, 2003) or right- (Saxe & Wexler, 2005) lateralised pSTS/TPJ activity, or bilateral activity (Saxe & Kanwisher, 2003). Developmental imaging studies of mentalising or social processing have reported greater right pSTS/TPJ activity in adults than in adolescents (Wang *et al.*, 2006; Blakemore *et al.*, 2007) and greater left pSTS/TPJ activity in adolescents than in adults (Wang *et al.*, 2006). More work is needed to elucidate whether left and right pSTS/TPJ play different cognitive roles in mentalising, what these roles may be, and whether they alter with age. Functional connectivity analyses conducted on existing datasets might shed light on the direct or modulatory roles of left and right pSTS/TPJ in mentalising tasks.

### Implications for the development of mentalising

An interpretation of the age-related decrease in connectivity between arMPFC and left pSTS/TPJ during social relative to basic emotions is that, in order to accomplish this task, adolescents require not only higher activity in arMPFC (as found in our conventional analysis) but also stronger co-activation of the mentalising system than do adults. This may be because the maturing network in adolescents is less efficient in accomplishing the task. Continuing synaptic elimination and axonal myelination (Yakovlev & Lecours, 1967; Huttenlocher, 1979; Huttenlocher *et al.*, 1982; Benes *et al.*, 1994; Huttenlocher & Dabholkar, 1997), and perhaps developing axonal calibre (Paus *et al.*, 2008) during adolescence, within regions of the brain involved in mentalising, may act to increase the efficiency of the system. Another (not mutually exclusive) possibility is that the shift in functional connectivity between arMPFC and left pSTS/TPJ with age is related to a change in the type of mentalising used in social cognition tasks such as these. Relative to adolescents, adults might employ a more automatic, less explicit, mentalising strategy. More naturalistic tests of mentalising, on which adolescents do not show ceiling performance, are needed to test this hypothesis, as well as studies combining measures of functional connectivity during mentalising tasks with static anatomical measures of grey and white matter volume and white matter integrity (e.g. voxel-based morphometry and diffusion-weighted imaging; Olesen *et al.*, 2003).

PPI analysis is used for investigating how an experimental manipulation modulates functional connectivity between brain regions, or alternatively how one brain region modulates the impact of an experimental manipulation on activity within a second brain region (Friston *et al.*, 1997). As PPI analysis tests for correlations, and not causal influences, a caveat is that it cannot inform as to the directionality of the relationships involved. In the current study, it could be the case that the social emotion task causes differences in connectivity between the mentalising regions, or instead that activity within arMPFC modulates the response of other mentalising regions to the social emotion task. The directionality of the functional connectivity reported here is unknown; whether arMPFC is influencing activity in the rest of the mentalising network, or vice versa, cannot be determined by PPI analysis.

## Conclusion

Several recent fMRI studies have shown evidence for the development during adolescence of activity within the mentalising system. However, to our knowledge, the development of functional connectivity within the mentalising system has not previously been studied in adolescents or in adults. This study represents an initial step in this direction. We have demonstrated that arMPFC shows functional connectivity with pSTS/TPJ and ATC during social relative to basic emotion processing, in both adolescents and adults. Future studies are needed to extend this finding, and to replicate our developmental result that functional connectivity between arMPFC and pSTS/TPJ during social relative to basic emotions decreases between adolescence and adulthood.

## Abbreviations

arMPFC: anterior rostral medial prefrontal cortex
ATC: anterior temporal cortex
fMRI: functional magnetic resonance imaging
FSIQ: full-scale IQ
PFC: prefrontal cortex
PPI: psychophysiological interaction
pSTS: posterior superior temporal sulcus
TPJ: temporo-parietal junction
